# Тактика ведения первичного гиперпаратиреоза c редкой локализацией эктопированного образования околощитовидной железы

**DOI:** 10.14341/probl13425

**Published:** 2024-04-01

**Authors:** Е. А. Абойшева, Е. С. Авсиевич, М. О. Корчагина, М. В. Дегтярев, Е. Е. Бибик, Д. Г. Бельцевич, Е. А. Пигарова, М. С. Шеремета

**Affiliations:** Национальный медицинский исследовательский центр эндокринологии; Национальный медицинский исследовательский центр эндокринологии; Национальный медицинский исследовательский центр эндокринологии; Национальный медицинский исследовательский центр эндокринологии; Национальный медицинский исследовательский центр эндокринологии; Национальный медицинский исследовательский центр эндокринологии; Национальный медицинский исследовательский центр эндокринологии; Национальный медицинский исследовательский центр эндокринологии

**Keywords:** первичный гиперпаратиреоз, околощитовидная железа, эктопия, гиперкальциемия, остеопороз, деносумаб, колекальциферол, 25-ОН витамин D, УЗИ, сцинтиграфия, ОФЭКТ/КТ, 4D-КТ, ПЭТ/КТ

## Abstract

Эктопическое расположение образований околощитовидных желез (ОЩЖ) у пациентов с первичным гиперпаратиреозом (ПГПТ) может затруднять проведение топической диагностики заболевания. Мы представляем клинический случай ПГПТ у пациентки 84 лет, обусловленный эктопированной аденомой ОЩЖ, локализованной позади правой внутренней яремной вены в проекции правой поднижнечелюстной слюнной железы. Отсутствие визуализации опухоли в течение длительного времени и высокий риск осложнений хирургического вмешательства требовали подбора консервативного лечения для контроля гиперкальциемии и коррекции осложнений ПГПТ. Ввиду тяжелого остеопороза и сопутствующей недостаточности витамина D к терапии деносумабом добавлен колекальциферол в насыщающих дозах под динамическим контролем показателей фосфорно-кальциевого обмена, что позволило достичь значимого снижения уровня ПТГ при сохранении нормокальциемии.

## Abstract (en)

Topical diagnosis can be severely complicated in patients with primary hyperparathyroidism (PHPT) due to the ectopic placement of parathyroid gland (PTG) formations. We report a clinical case of PHPT in an 84-year-old patient caused by ectopic PTG adenoma located behind the right internal jugular vein at the level of the right submandibular gland. The impossibility of surgery for a long time due to the absence of a topical diagnosis has necessitated conservative treatment was required to get the hypercalcemia under control. In view of the concomitant deficiency of vitamin D, an attempt was made to use therapy with saturating doses of cholecalciferol under dynamic monitoring of the indicators of phosphorus-calcium metabolism, which allowed to achieve a significant decrease in PTH levels while maintaining normocalcemia.

## АКТУАЛЬНОСТЬ

Первичный гиперпаратиреоз (ПГПТ) — эндокринное заболевание, характеризующееся избыточной секрецией паратиреоидного гормона (ПТГ) при верхне-нормальном или повышенном уровне кальция крови вследствие первичной патологии околощитовидных желез (ОЩЖ). Гиперпаратиреоз относится к одной из наиболее распространенных эндокринопатий, занимая третье место после сахарного диабета (СД) и заболеваний щитовидной железы (ЩЖ). В общей популяции распространенность ПГПТ составляет в среднем около 0,86–1%. [1]. Согласно данным Российского регистра пациентов с ПГПТ, выявляемость этого заболевания в Российской Федерации составляет 1,3 на 100 тыс. населения [2].

В результате нарушения регуляции и чрезмерной секреции ПТГ одной или несколькими патологически измененными ОЩЖ происходит повышение уровня кальция (Са) крови. Это обусловлено усилением его реабсорбции в почечных канальцах, стимуляцией опосредованной остеокластами резорбции кости и увеличением почечного синтеза 1,25(OH)2D3, который в свою очередь способствует усилению всасывания Са и фосфатов в кишечнике. Классические проявления ПГПТ, как правило, выражаются в поражении костной системы и почек, при этом у пациентов наблюдаются снижение минеральной плотности костной ткани (МПК), низкоэнергетические переломы, нефролитиаз, нефрокальциноз, в ряде случаев — развитие хронической почечной недостаточности [3].

ПГПТ может быть компонентом наследственных синдромов, таких как синдромы множественных эндокринных неоплазий 1, 2А и 4 типов и синдром гиперпаратиреоза с опухолью челюсти (hyperparathyroidism-jaw tumor syndrome — HPT-JT), но чаще является изолированным спорадическим заболеванием [4].

В 80–90% наблюдений причиной спорадического ПГПТ становится солитарная аденома ОЩЖ, реже — полигландулярное поражение или изменение ткани железы злокачественного характера (в 10–15% — гиперплазия четырех ОЩЖ, в 5% — множественные аденомы и менее чем в 1% случаев — рак ОЩЖ) [5].

У человека обычно присутствует 4 парные ОЩЖ (2 верхние и 2 нижние), примерно у 10% населения эмбрионально развиваются только 2–3 железы, у 5% отмечается 5 и более ОЩЖ. Наибольшее количество ОЩЖ, по данным секционных исследований, достигает 12 [6][7].

Верхние ОЩЖ берут начало из четвертого глоточного кармана, в процессе эмбрионального развития опускаются вместе с ЩЖ и в итоге остаются возле перстнещитовидного хряща, позади ЩЖ и возвратного гортанного нерва. Нижние ОЩЖ развиваются из третьего глоточного кармана, опускаются вместе с тимусом и к моменту рождения обычно находятся вблизи нижней границы ЩЖ, впереди возвратного гортанного нерва [8][9].

При нарушении эмбриональной миграции железы могут либо чрезмерно опуститься, либо остаться на месте в области первичных зачатков. Большинство аденом ОЩЖ локализуются в типичном месте, однако в 22–35% случаев располагаются эктопически [8][10].

К возможным атипичным локализациям относятся тимус (38%), ретроэзофагеальная область (31%), ЩЖ (18%), средостение (6%) и влагалище сонной артерии (3%) [11]. Нижние ОЩЖ из-за более длинного пути миграции при эмбриональном развитии имеют большую вариабельность эктопии, чем верхние, и могут располагаться в пространстве от нижней челюсти до верхнего средостения [12][13]. В некоторых описанных случаях сообщалось о чрезвычайно редких и необычных местах локализации, таких как перикард [14].

Ввиду трудностей проведения топической диагностики и хирургического лечения, образования эктопированных ОЩЖ могут стать причиной персистирующего ПГПТ с тяжелыми клиническими проявлениями [15][16].

Мы представляем случай пациентки с длительным течением ПГПТ вследствие атипично расположенного образования ОЩЖ позади правой внутренней яремной вены на уровне правой поднижнечелюстной слюнной железы. Данный случай наглядно иллюстрирует проблему диагностики и определения тактики ведения пациентов с эктопированно расположенным образованием ОЩЖ.

## ОПИСАНИЕ КЛИНИЧЕСКОГО СЛУЧАЯ

У пациентки У. впервые диагностирован ПГПТ в 2011 г. в возрасте 74 лет. В течение заболевания отмечалось развитие выраженной гиперкальциемии и остеопороза. С 2017 г. инициирована терапия деносумабом 60 мг 1 раз в полгода. Эндокринологом по месту жительства рекомендовано хирургическое лечение, однако в течение длительного периода при использовании различных методов топической диагностики (ультразвукового исследования (УЗИ), компьютерной томографии (КТ) шеи с контрастом, планарной сцинтиграфии), проводившихся в различных учреждениях, не удавалось обнаружить образование ОЩЖ.

С февраля 2021 г. для коррекции гиперкальциемии к терапии также добавлен цинакальцет 30 мг в сутки с увеличением дозы до 90 мг. Однако препарат отменили через 9 месяцев терапии из-за развития гастроинтестинальных побочных эффектов (динамика лабораторных показателей изложена в таблице 1).

**Table:** Таблица 1. Результаты лабораторных исследований пациентки в амбулаторных условиях

Дата	Са общий, ммоль/л (2,2–2,65)	Са, скорректированный на альбумин, ммоль/л (2,2–2,65)	ПТГ, пмоль/л (1,7–6,4)
Ноябрь 2020 — Инъекция деносумаба
12.2020	3,03		29,5	
02.2021			41,78	ЦИНАКАЛЬЦЕТ
Май 2021 — Инъекция деносумаба
06.2021	2,5		25,5
09.2021	2,96	2,88	18,02
Ноябрь 2021 — Инъекция деносумаба
11.2021	2,46	2,38	134,6

В декабре 2021 г. были проведены сцинтиграфия ОЩЖ с 99mTc-технетрилом и однофотонная эмиссионная КТ, совмещенная с рентгеновской КТ (ОФЭКТ/КТ) в ГНЦ РФ ФГБУ «НМИЦ эндокринологии» Минздрава России (далее — НМИЦ эндокринологии), а также мультиспиральная КТ шеи с контрастным усилением. Именно при помощи ОФЭКТ/КТ впервые было выявлено атипично высоко расположенное образование с признаками интенсивного накопления радиофармпрепарата размерами 18х11х33 мм справа, позади правой внутренней яремной вены, медиальнее m. sternocleidomastoideus на уровне правой поднижнечелюстной слюнной железы (рис. 1).

**Figure:**
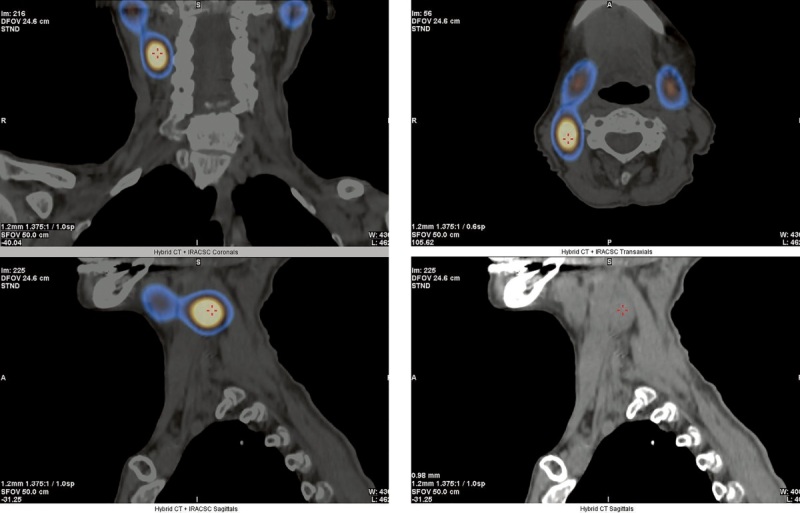
Рисунок 1. ОФЭКТ/КТ с 99mTc-технетрилом от декабря 2021 г.Красными метками отмечено образование ОЩЖ.

Объемное образование подтверждено в ходе проведения мультиспиральной компьютерной томографии (МСКТ) шеи с контрастным усилением (рис. 2).

**Figure:**
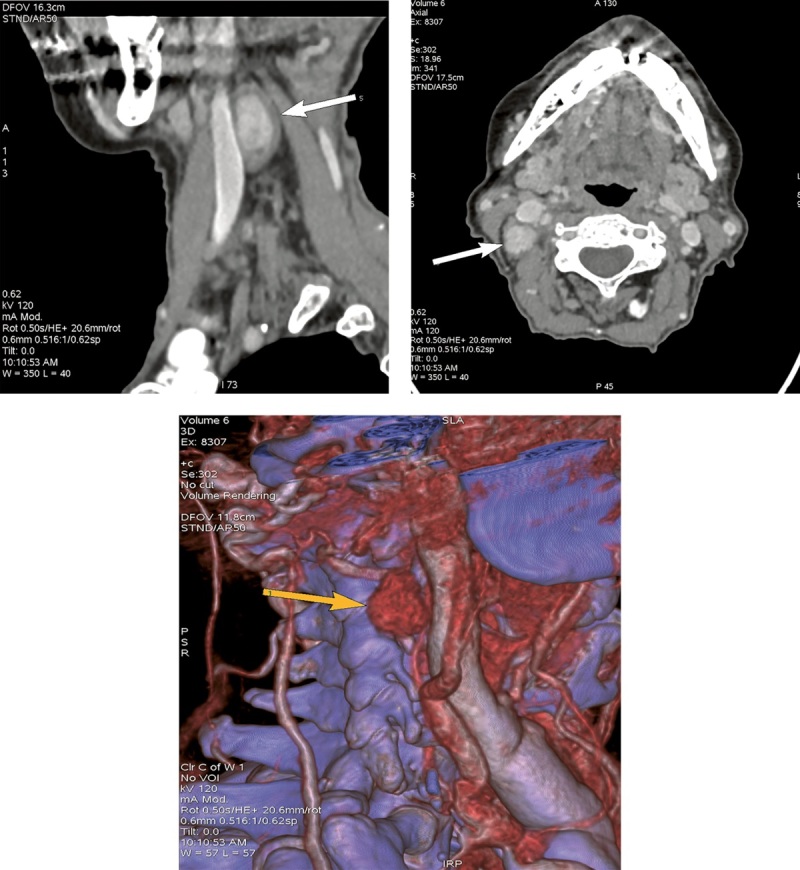
Рисунок 2. МСКТ шеи с контрастным усилением от декабря 2021 г.Стрелками отмечено атипично расположенное образование ОЩЖ.

Пациентка была осмотрена эндокринным хирургом, при пальпации в проекции образования отмечался кашель, что могло указывать на расположение опухоли вблизи возвратного гортанного нерва.

В декабре 2021 г. в возрасте 84 лет пациентка госпитализирована в отделение патологии околощитовидных желез и нарушений минерального обмена НМИЦ эндокринологии с жалобами на снижение массы тела на 6 кг за год, слабость, боли в спине, уменьшение в росте на 8 см, периодические эпизоды повышения артериального давления до 200/100 мм рт.ст. При осмотре отмечалась сниженная масса тела — 46 кг при росте 153 см (ИМТ — 19,7 кг/м²).

По данным лабораторных исследований, на фоне инъекции деносумаба за 1 мес. до госпитализации выявлено повышение ПТГ до 1599 пг/мл (15–65) на фоне высоко-нормального уровня кальция крови (кальций общий — 2,48 ммоль/л, кальций скорректированный на альбумин — 2,40 ммоль/л). При исследовании суточной мочи выявлена гипокальциурия — 0,65 ммоль/л (2,5–8), вероятно, вследствие антирезорбтивной терапии. Фильтрационная функция почек сохранялась удовлетворительной (креатинин — 59,6 мкмоль/л, СКФ по формуле CKD-EPI — 80 мл/мин/1,73 м²).

По результатам рентгеновской денситометрии (dual-energy X-ray absorptiometry — DXA) подтвержден выраженный остеопороз: снижение МПК поясничных позвонков до -4,8 SD, лучевой кости до -4,8 SD, бедренной кости до -3,5 SD по Т-критерию. При рентгенографии грудного и поясничного отделов позвоночника компрессионных переломов не выявлено. По результатам УЗИ почек, данных за нефролитиаз, нефрокальциноз не получено.

Для оценки периоперационных рисков хирургического лечения проведена диагностика состояния сердечно-сосудистой системы. У пациентки осложненный кардиологический анамнез: в 2005 г. выполнено стентирование правой коронарной артерии, в 2018 г. перенесла инфаркт миокарда, при дальнейшем обследовании выявлен рестеноз в стенте до 50%. Для уточнения состояния больной был запланирован тредмил-тест, однако выполнить исследование не удалось по техническим причинам, ввиду чего рекомендовано стресс-ЭХО-КГ с добутамином.

В связи с наличием тяжелой сопутствующей патологии, пожилым возрастом возникла необходимость в поиске оптимального варианта консервативного лечения. С учетом недостаточности витамина D (25(ОН)D 26,6 нг/мл) и нормокальциемии, по данным биохимического анализа крови в течение госпитализации, был инициирован прием насыщающей дозы колекальциферола (7000 МЕ ежедневно в течение 6 дней). По данным контрольного анализа крови, уровень ПТГ составил 350,2 пг/мл, отмечено увеличение уровня общего кальция до 2,58 ммоль/л, показатель кальция, скорректированного на альбумин — в пределах референсных значений (2,48 ммоль/л).

Пациентке рекомендовано продолжить прием колекальциферола для достижения целевых значений уровня 25(ОН) витамина D крови, а также терапию деносумабом.

## ОБСУЖДЕНИЕ

В представленном клиническом случае эктопическое расположение образования ОЩЖ привело к значимо отсроченному определению топического диагноза, необходимого для проведения паратиреоидэктомии.

Как правило, УЗИ — первый этап топической диагностики при ПГПТ. Чувствительность данного метода исследования в отношении образований ОЩЖ составляет от 55 до 87% (при солитарных образованиях достигает 91%) и снижается при сопутствующем узловом зобе. Оценка эктопированных ОЩЖ на УЗИ может быть весьма затруднена, а при некоторых локализациях — невозможна [17].

Радионуклидное исследование — второй этап визуализации образований ОЩЖ. Длительное время стандартной методикой поиска измененных ОЩЖ была двухмерная планарная сцинтиграфия, в настоящее время все чаще используется сочетание трехмерной однофотонной эмиссионной компьютерной томографии (ОФЭКТ) и гибридной технологии ОФЭКТ/КТ. Трехмерное сканирование лучше выявляет труднодоступные для диагностики аденомы эктопированных ОЩЖ и небольшие образования [18–20]. Гибридные технологии позволяют дифференцировать поражения ОЩЖ от других источников поглощения 99mTc-технетрила, включая узлы ЩЖ и шейные лимфатические узлы, а также очаги физиологического поглощения РФП [21]. Ложноположительные результаты могут возникать при многоузловом зобе, тиреоидите и лимфаденопатии. В представленном нами клиническом случае именно ОФЭКТ, совмещенная с КТ, позволила впервые обнаружить образование ОЩЖ.

В случае отсутствия четкой визуализации патологической ОЩЖ после проведения методов первой линии рекомендуется выполнение дополнительных исследований. Диагностические исследования второй линии включают в себя КТ с внутривенным (в/в) контрастированием, позитронно-эмиссионную томографию (ПЭТ) с радиофармацевтическим препаратом, совмещенную с компьютерной томографией (ПЭТ/КТ) или с магнитно-резонансной томографией (ПЭТ/МРТ), МРТ с в/в контрастированием, четырехмерную КТ (4D-КТ) [22]. В ряде работ 4D-КТ продемонстрировала более высокую чувствительность, чем сканирование с 99mTc-технетрилом и УЗИ. 4D-КТ может стать полезным методом диагностики для пациентов с полигландулярным поражением или при эктопии ОЩЖ. Основные недостатки метода — значительная лучевая нагрузка, высокая частота ложноположительных результатов, малодоступность и высокая стоимость [23][24]. ПЭТ, как правило, используется у пациентов со злокачественными образованиями ОЩЖ для оценки распространенности метастатического процесса. К преимуществам ПЭТ/КТ с 18F-холином относят более короткое время визуализации, более высокое пространственное разрешение и меньшую дозу облучения (2,8 мЗв), чем при ОФЭКТ/КТ с 99mTc-технетрилом (6,8 мЗв). Однако применение данного метода ограничено из-за высокой стоимости и низкой доступности [25][26]. Чувствительность ПЭТ/КТ с 11С-метионином при образованиях ОЩЖ может достигать 91%, ПЭТ/КТ с 18F-холином — 96% [27][28].

Другие методики, такие как селективный забор крови и интраоперационное введение 0,1% водного раствора метиленового синего в нижнюю щитовидную артерию, не получили широкого распространения [29].

У пациентов с ПГПТ для медикаментозной коррекции гиперкальциемии широкое применение получили цинакальцет, деносумаб и бисфосфонаты [18]. Терапевтическая тактика в отношении назначения препаратов витамина D часто разнится.

По данным исследований, распространенность недостаточности витамина D среди пациентов с ПГПТ выше, чем в общей популяции. Вероятно, высокий уровень ПТГ усиливает превращение 25(ОН) витамина D в активный 1,25(ОН)2 витамин D, а также приводит к инактивации витамина D посредством усиленного печеночного метаболизма [30–32].

По результатам проведенных исследований опасения относительно провокации гиперкальциемии у пациентов с ПГПТ при восполнении дефицита витамина D не оправдывались. Так, по данным двух метаанализов Shan VN и соавт. и Song A и соавт., терапия препаратами витамина D у пациентов с ПГПТ и дефицитом витамина D значительно снижает уровень ПТГ, не вызывая гиперкальциемии и гиперкальциурии [33][34]. Согласно Shan VN, частота выраженной гиперкальциемии (более 3 ммоль/л) составила всего 2,2% [34]. Стоит отметить, что в исследованиях в используемых схемах лечения наблюдались значительные различия в длительности, дозах и формах применяемых препаратов.

По данным двойного слепого рандомизированного контролируемого исследования 6-месячный курс приема колекальциферола у пациентов с ПГПТ приводил к значимому повышению уровня витамина D, снижению уровня ПТГ (на 17%), приросту МПК (на 2,5%) при стабильном уровне кальция в сыворотке крови и моче [35]. Сходные данные были получены при исследовании влияния терапии эргокальциферолом: на фоне лечения не было обнаружено значительного увеличения уровня кальция крови или соотношения кальций/креатинин в моче при незначительном снижении уровня ПТГ [36]. Необходимо отметить, что практически во всех исследованиях пациентам назначалась терапия препаратами витамина D в поддерживающей дозе [18].

В представленном нами клиническом случае планарная сцинтиграфия не позволила локализовать аденому ОЩЖ, поскольку аденома располагалась рядом с поднижнечелюстной слюнной железой и при данном методе исследования не дифференцировалась на фоне физиологического накопления 99mTc-технетрила в слюнной железе. И только объемное изображение ОФЭКТ/КТ позволило обнаружить аденому ОЩЖ. В данном клиническом случае пожилой возраст пациентки, эктопическое расположение образования, расположение опухоли ОЩЖ вблизи возвратного гортанного нерва, потенциально осложняющее хирургическую тактику, а также наличие тяжелой сопутствующей кардиоваскулярной патологии значимо повышали операционные риски. В силу отсутствия топических данных на протяжении длительного времени своевременное радикальное хирургическое лечение не представлялось возможным. Вышеуказанные факторы, а также возможность поддержания нормокальциемии на фоне медикаментозной терапии послужили причиной выбора консервативной тактики лечения пациентки.

Ввиду высоких значений ПТГ, выраженной гиперкальциемии и размера образования более 3 см в наибольшем измерении сохранялась настороженность в отношении злокачественного поражения ОЩЖ. Применение колекальциферола в насыщающей дозе позволило добиться значимого снижения ПТГ при сохранении нормокальциемии, что свидетельствует о вероятном смешанном генезе гиперпаратиреоза (первичном и вторичном на фоне недостаточности 25 ОН витамина D, терапии антирезорбтивными препаратами). В настоящее время пациентка продолжает терапию колекальциферолом в насыщающей дозе с последующим переходом на поддерживающую под динамическим контролем показателей фосфорно-кальциевого обмена.

## ЗАКЛЮЧЕНИЕ

Представленный клинический случай демонстрирует сложности, возникающие при проведении топической диагностики у пациентов с атипичным расположением образований ОЩЖ. Применение современных методов инструментальной диагностики дает возможность четко установить диагноз и определить дальнейшую тактику ведения пациента, что особенно важно при эктопированных образованиях ОЩЖ. Однако стоит отметить, что в сложных клинических случаях проведение топической диагностики необходимо выполнять в специализированных медицинских учреждениях.

Проведение терапии колекальциферолом под динамическим контролем показателей фосфорно-кальциевого обмена может способствовать положительной динамике состояния у пациентов с ПГПТ, имеющих сопутствующий дефицит или недостаточность витамина D, снижение функции почек и другие состояния, способные провоцировать вторичное повышение ПТГ.

## ДОПОЛНИТЕЛЬНАЯ ИНФОРМАЦИЯ

Источники финансирования. Работа выполнена по инициативе авторов без привлечения финансирования.

Конфликт интересов. Авторы декларируют отсутствие явных и потенциальных конфликтов интересов, связанных с содержанием настоящей статьи.

Участие авторов. Авторы декларируют соответствие своего авторства международным критериям ICMJE. Все авторы внесли равный вклад в подготовку статьи и одобрили ее окончательный вариант.

Согласие пациента. Пациент добровольно подписал информированное согласие на публикацию персональной медицинской информации в обезличенной форме в журнале «Проблемы эндокринологии».
